# Ongoing transcatheter aortic valve implantation (TAVI) practice amidst a global COVID-19 crisis: nurse-led analgesia for transfemoral TAVI

**DOI:** 10.1007/s12471-020-01472-4

**Published:** 2020-07-13

**Authors:** J. Vendrik, J. de Boer, W. Zwiers, S. A. van Gilst, M. Holierook, E. V. Chekanova, J. S. Henriques, J. Baan Jr.

**Affiliations:** Department of Cardiology, Heart Centre, Amsterdam UMC, University of Amsterdam, Amsterdam, The Netherlands

**Keywords:** Minimalist transcatheter aortic valve implantation, Catheterisation laboratory, Local analgesia, Transfemoral transcatheter aortic valve implantation, Anaesthesiologist, Procedural complications

## Abstract

The current coronavirus disease 2019 (COVID-19) crisis has led to a relative unavailability of anaesthesiological support for non-acute cardiac care. Currently, transfemoral transcatheter aortic valve implantation (TF-TAVI) is predominantly performed as an elective catheterisation laboratory (cath lab) procedure. Hence, the performance of TAVI could come to a halt amidst the COVID-19 crisis. Our study population comprised 90 patients treated with TF-TAVI, with local analgesia performed by our dedicated cath lab nurses. The patients had a mean age of 80 ± 5 years and 59% were male, with a predicted surgical risk of 2.2 ± 0.9/3.1 ± 2.4% (Society of Thoracic Surgeons Predicted Risk of Mortality [STS-PROM] score/EuroSCORE II), depicting a contemporary, lower-risk population. The composite endpoint of device success (Valve Academic Research Consortium [VARC]-2) was reached in all patients. No patients showed more than mild paravalvular leakage (3/90, 3.3%). Overall, intravenous medication was sparsely used during the procedure, with 48 of the 90 (53%) patients receiving no unplanned intravenous medication. There was neither procedural nor in-hospital mortality. The performance of TF-TAVI using local analgesia only, managed by a dedicated nurse instead of an anaesthesiologist, was shown to be feasible and safe in a selected group of patients. This strategy may (temporarily) eliminate the need for an anaesthesiologist to be present in the cath lab and enables ongoing TAVI treatment amidst the global COVID-19 crisis.

## Introduction

Transcatheter aortic valve implantation (TAVI) is a well-established treatment for aortic valve stenosis which has been widely adopted and has evolved into a minimalistic, relatively low-risk procedure for the majority of patients. Using local analgesia only, instead of conscious sedation or general anaesthesia, minimalises the invasive nature of the procedure and has been shown to lower the incidence of postoperative delirium and to decrease the duration of hospitalisation [1–3]. Left untreated, symptomatic severe aortic stenosis has a dismal prognosis.

The current COVID-19 crisis has led to a relative unavailability of anaesthesiological support for non-acute cardiac care. In current practice, transfemoral (TF)-TAVI is predominantly performed as an elective catheterisation laboratory (cath lab) procedure. Hence, the performance of TAVI could come to a halt amidst the COVID-19 crisis. With this study, we aim to show the safety and feasibility of TF-TAVI with nurse-led local analgesia, possibly eliminating the need for an anaesthesiologist to be present in the cath lab.

## Methods

**Fig. 1:**
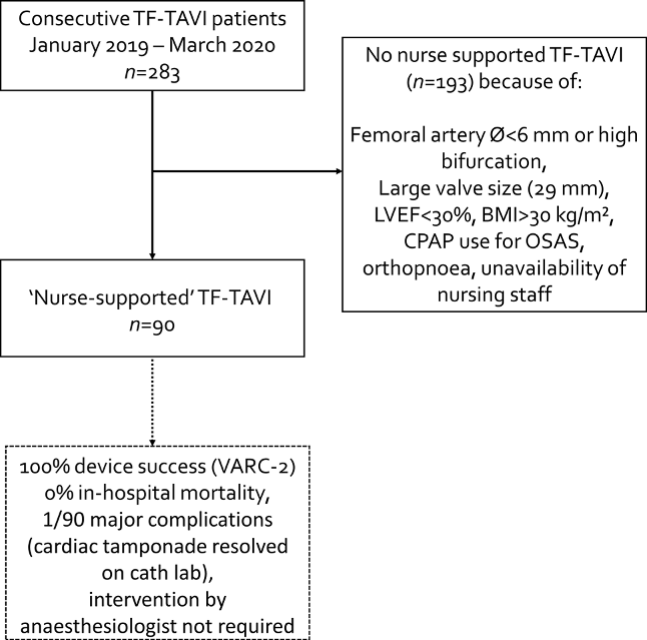
Flowchart of patient selection and procedural outcome. (*TF-TAVI* transfemoral transcatheter aortic valve implantation, *LVEF* left ventricular ejection fraction, *BMI* body mass index, *CPAP* continuous positive airway pressure, *OSAS* obstructive sleep apnoea syndrome, *VARC* Valve Academic Research Consortium)

The study population comprised all consecutive patients receiving nurse-supported TF-TAVI from January 2019 to March 2020 at the Amsterdam University Medical Centre (Amsterdam UMC, location AMC) (Fig. 1). We selected eligible patients for nurse-supported TAVI via a stepwise approach. First, we performed a risk analysis based on our extensive experience in our prospective database. We found that the risk of complications that would require the immediate support of an anaesthesiologist to achieve an acceptable outcome was 0.23%. Thereafter, we formulated a stepwise implementation plan. Cath lab nurses with profound TAVI experience were additionally trained in the use of anaesthesia, noradrenaline and rapid pacing. Initially, during all procedures an anaesthesiologist was physically present in an adjacent room, as a back-up. Exclusion criteria were initially more stringent and have become less stringent over time. The initial exclusion criteria are shown in Fig. 1. The Institutional Review Board of the Amsterdam UMC granted this study a waiver.

## Results

Ninety patients were treated with TF-TAVI supported by our dedicated cath lab nurses. The patients had a mean age of 80 ± 5 years and 59% were male, with a predicted surgical risk of 2.2 ± 0.9/3.1 ± 2.4% (Society of Thoracic Surgeons Predicted Risk of Mortality [STS-PROM]/EuroSCORE II), depicting a contemporary, lower-risk population. All patients were treated with the SAPIEN 3 aortic valve prosthesis (Edwards Lifesciences, Irvine, CA, USA), implanted using local analgesia only. Forty-four (51%) prostheses were implanted directly (without predilation). Postdilation was performed in only 5 of the 90 (5.5%) cases. Mean procedural time was 38 ± 10 min, of which 11 ± 4 min were fluoroscopy time. The composite endpoint of device success (Valve Academic Research Consortium [VARC]-2) was reached in all patients. No patients showed more than mild paravalvular leakage (3/90, 3.3%). Overall, intravenous medication was sparsely used during the procedure. Intravenous paracetamol or fentanyl was used in 28 (31%) and 14 (16%) of the 90 patients, respectively; 12 (13%) received noradrenaline and in 7 (7.8%) cases nitroglycerin was administered. Of the 90 patients, 48 (53%) received no unplanned intravenous medication. This distribution did not differ from that of procedures supported by anaesthesiologists, in our experience.

There was neither procedural nor in-hospital mortality. One major procedural complication occurred. This patient suffered from cardiac tamponade, which was fully resolved by immediate pericardiocentesis, without anaesthesiological support, after which the patient had an uneventful recovery.

## Discussion

This small, single-centre, prospective real-life registry performed in an experienced, high-volume centre shows the safety and feasibility of TF-TAVI with nurse-led analgesia in a selected group of patients. Intervention by an anaesthesiologist was not required in this cohort.

TAVI has become a much lower-risk procedure, allowing the majority of cases to be performed without an anaesthesiologist present in the cath lab. Although TAVI cannot be compared with a regular percutaneous coronary intervention (PCI), it is noteworthy that PCIs were also initially performed with the support of an anaesthesiologist [4]. This is no longer the case, as the technique and procedure have gradually been minimalised. Nevertheless, such a dedicated nurse programme should be preferably initiated with good risk assessment, training, planning and evaluation. Our procedures were performed in a tertiary heart centre with extensive experience (±1500 cases without transoesophageal echocardiography guidance and without general anaesthesia since 2010) and all equipment (such as echocardiography machine and peripheral left ventricular support devices), the required staff and an operating theatre available on demand. Hence, extrapolation to other (less experienced or less well equipped) centres should be done with extreme caution.

TF-TAVI with nurse-led analgesia will evidently facilitate easier procedural planning, thereby shortening the potentially hazardous waiting list for the procedure in regular clinical care. Right now, during the global COVID-19 crisis, this strategy may (temporarily) enable ongoing TAVI treatment and therefore may prevent non-COVID-related deaths. On the other hand, the hospitalisation of frail, elderly TAVI patients could introduce an increased risk of COVID-19 infection. Thus, careful patient selection, considering the changed risk-benefit ratio in the current pandemic, is required. Mentias et al. [5] recently proposed an algorithm for the timing of TAVI based on the health status of the patients and following the urgency of the procedure, which in our opinion could be used as a guideline in these unusual times.

Roughly 3 out of 4 patients were deemed eligible for nurse-supported TF-TAVI by our heart team. Yet, predominantly because of the unavailability of trained nursing staff, only 34% of all procedures in our cohort were performed with nurse support. From March 2020 onwards, after the COVID-19 crisis hit the Netherlands and subsequent government guidelines were introduced, we have performed all TF-TAVIs with nurse-led analgesia and without the need for an anaesthesiologist to be present.

## Conclusion

The performance of transfemoral TAVI using local analgesia only, supported by a dedicated nurse instead of an anaesthesiologist, is feasible and safe in a selected group of patients. This strategy may temporarily eliminate the need for an anaesthesiologist to be present in the cath lab, and enables ongoing TAVI treatment amidst the global COVID-19 crisis.
